# Deterioration in Quality of Life among COVID-19 Survivors: Population-Based Cohort Study

**DOI:** 10.3390/jpm14060569

**Published:** 2024-05-26

**Authors:** Tak Kyu Oh, In-Ae Song

**Affiliations:** 1 Department of Anesthesiology and Pain Medicine, Seoul National University Bundang Hospital, Seongnam 13620, Republic of Korea; 66034@snubh.org; 2 Department of Anesthesiology and Pain Medicine, College of Medicine, Seoul National University, Seoul 01811, Republic of Korea

**Keywords:** public health, COVID-19, infection, epidemiology, population

## Abstract

We aimed to examine the prevalence of, and factors associated with, quality of life (QOL) worsening among coronavirus disease 2019 (COVID-19) survivors. This population-based retrospective cohort study used data from the Korea Disease Control and Prevention Agency and the National Health Insurance Service in South Korea. A total of 325,666 COVID-19 survivors were included in this study. Among them, 106,091 (32.6%) survivors experienced worsening QOL after COVID-19. Specifically, 21,223 (6.5%) participants experienced job loss, 94,556 (29.0%) experienced decreased household income, and 559 (0.2%) acquired new disabilities. In the multivariable logistic regression model, living in rural areas (odds ratio [OR]: 1.02; 95% confidence interval [CI]: 1.01, 1.04; *p* = 0.009), intensive care unit admission (OR: 1.08, 95% CI: 1.02, 1.15; *p* = 0.028), and increase in self-payment by 100 USD (OR: 1.02, 95% CI: 1.02, 1.02; *p* < 0.001) were associated with increased QOL worsening after COVID-19. Old age (OR: 0.99, 95% CI: 0.98, 0.99; *p* < 0.001), first vaccination (OR: 0.89, 95% CI: 0.86, 0.93; *p* < 0.001), and second vaccination (OR: 0.95, 95% CI: 0.93, 0.96; *p* < 0.001) were associated with decreased QOL worsening after COVID-19. Approximately one-third of COVID-19 survivors in South Korea who were admitted to hospitals or monitoring centers experienced QOL worsening.

## 1. Introduction

Coronavirus disease 2019 (COVID-19) was declared a pandemic by the World Health Organization on 11 March 2020 [1], and the COVID-19 crisis has been considered as the most important public health issue during the pandemic [2]. Since vaccination for COVID-19 began on 8 December 2020 [3,4], COVID-19 recently transitioned from being a pandemic to being an endemic [5]. The global COVID-19 experience will affect health policies, new health priorities, approaches, and new agendas in the future as a major public health issue [6].

Many patients with COVID-19 required admission to the hospital for treatment [7], and survivors who are discharged from the hospital could suffer a deterioration in quality of life (QOL) [8]. Hospitalized COVID-19 survivors report persistent symptoms, particularly fatigue and breathlessness, with low QOL scores [9]. This is because COVID-19 infection could affect or damage multiple body systems, such as the cardiac, neurological, cognitive, and mental health systems, in addition to pulmonary complication, which requires that COVID-19 survivors undergo rehabilitation [10].

Previous studies have reported on the QOL of COVID-19 survivors regarding social function, physical role, mental health, pain, activity change, and discomfort [11]. Worsening QOL among COVID-19 survivors is an important health issue in China [12] and the United States [13]. However, most of these previous studies did not assess the QOL of COVID-19 survivors regarding unemployment, economic status, and newly acquired disability. Job maintenance, household income, and physical functioning are critical for people with COVID-19 to resume their daily lives. Thus, it is necessary to know the type of QOL worsening that occurs in COVID-19 survivors socioeconomically to facilitate the development of social policy support for them. Moreover, the newly acquired disability could reflect long-term functional impairment associated with surviving COVID-19 after hospital discharge.

Therefore, we aimed to examine the prevalence and associated factors of QOL worsening among COVID-19 survivors regarding unemployment, decreased household income, and newly acquired disability using a nationwide registration database in South Korea.

## 2. Materials and Methods

### 2.1. Study Design and Setting

This population-based retrospective cohort study followed the Strengthening the Reporting of Observational Studies in Epidemiology guidelines [14]. The Institutional Review Board (IRB) of Seoul National University Bundang Hospital reviewed our study protocol and exempted it from further deliberation after confirming from the study protocol that we used public data available to all researchers (IRB number: X-2205-758-901). Due to the retrospective nature of the data and anonymous nature of the analysis, the IRB waived the need for informed consent.

### 2.2. Data Source

For policy and academic research in South Korea, we utilized the databases of the Korea Disease Control and Prevention Agency (KDCA) and the National Health Insurance Service (NHIS). The research number used for this investigation was KDCA-NHIS-2022-1-489. Initially, the KCDA provided the NHIS with information pertaining to patients identified as having COVID-19 through polymerase chain reaction (PCR) testing between 8 October 2020 and 31 December 2021. Age, sex, date of COVID-19 diagnosis via PCR testing, date of mortality, date of vaccination, route of infection, and cause of infection were among the variables extracted from the KCDA database. The routes of infection were categorized as either domestic or foreign. The causes of infection were classified into the following six groups: (1) inflow from foreign countries, (2) contact with person-related inflow from foreign countries, (3) outbreak in hospitals or nursing care centers, (4) outbreak in local communities, (5) contact with a COVID-19 confirmed patient, and (6) unknown cause. As the route of transmission of COVID-19 is most likely through droplets, transmission may be influenced by the socioeconomic status of the patients. The NHIS furnished, until 31 March 2022, information pertaining to prescriptions for procedures or medications, demographic and socioeconomic statuses, and all disease diagnoses detailed using International Classification of Diseases (ICD)-10 codes, in addition to data obtained from the KDCA. Thus, we used a database derived from the collaboration between the KDCA and NHIS.

#### Treatment Pattern of COVID-19

Hospitalization is the standard course of action for individuals infected with COVID-19 who present with severe symptoms, including pneumonia. If they exhibit minimal or no symptoms, they are sequestered and monitored closely in monitoring centers administered by the government. If the patients with COVID-19 in the government-managed centers develop severe symptoms, they are immediately transferred to a hospital for proper treatment. However, if young patients without an underlying disease are diagnosed with COVID-19, they are isolated at home. The government pays for the entire cost of treatment for patients diagnosed with COVID-19 that are covered by insurance, excluding some non-insured treatments.

### 2.3. Study Population: COVID-19 Survivors

**Figure 1:**
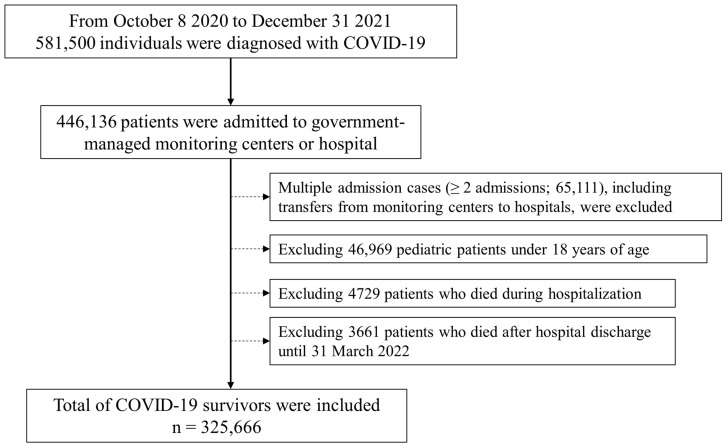
Flowchart of the selection of COVID-19 survivors. COVID-19, coronavirus disease 2019.

From 8 October 2020 to 31 December 2021, 581,500 individuals were diagnosed with COVID-19 by PCR testing. Among them, 446,136 patients were admitted to government-managed monitoring centers or hospitals. Multiple admission cases (≥2 admissions; 65,111), including transfers from monitoring centers to hospitals, were excluded. After excluding 46,969 pediatric patients under 18 years of age, 4729 patients who died during hospitalization, and 3661 patients who died after hospital discharge until 31 March 2022, a total of 325,666 COVID-19 survivors were included in the analysis (Figure 1).

### 2.4. Quality of Life Assessment

QOL was determined using the following three factors: unemployment, household income level, and disability, and QOL worsening was defined as the occurrence of unemployment, decreased household income, and/or newly acquired disability in COVID-19 survivors. Even if only one of these three factors occurred, it was considered as QOL worsening. Those who experienced any QOL worsening were considered the QOL worsening group, while the remaining patients were considered the no QOL worsening group.

As the KDCA-NHIS database on the study patients diagnosed with COVID-19 by PCR testing from 8 October 2020 to 31 December 2021 provides data until 31 March 2022, the QOL worsening of COVID-19 survivors was evaluated on 31 March 2022 and compared with their QOL in the year when they were diagnosed with COVID-19.

In the context of this research, self-employed individuals were classified as employed. Two methods of calculating household income level were utilized: employee insured and self-employed insured. The insurance premium for individuals who were self-employed was established based on factors such as the rate of economic participation, income, property, and living standards, while that for employee-insured individuals was determined by income. Nevertheless, individuals who encountered financial hardships or were unable to afford insurance premiums were granted eligibility for the medical assistance program. Using quartile ratios, household income was categorized into four distinct groups, with Q4 representing the highest and Q1 representing the lowest, in addition to the medical aid program group. All individuals with disabilities in South Korea are enrolled in the NHIS database in order to be eligible for a range of benefits. Physical and brain lesion disabilities; visual disturbances; hearing and speech disabilities; autism; intellectual, mental, renal, heart, and respiratory disorders; facial disfigurements; hepatopathies; intestinal and urinary fistulae; and epilepsy comprised the fifteen categories of disabilities. According to severity, the degrees of disability were categorized into two groups: severe disability and mild-to-moderate disability. The disability was accurately diagnosed and determined in strict adherence to the prescribed definition by a physician who specialized in that particular field. Predominant in the determination of disability was the extent to which it impeded the execution of routine activities of daily living. As the NHIS is the only public insurance system in South Korea, all individuals in South Korea are registered in the NHIS database; therefore, QOL could be evaluated in the South Korean population. In addition, any foreigner or overseas Korean who has stayed in South Korea for six or more months are required to statutorily subscribe to the NHIS.

### 2.5. Other Analyzed Variables

The residential areas affected by the COVID-19 outbreak were categorized as either rural (excluding Seoul and other major cities) or urban (including the rest of the country). As shown in Table S1, the comorbidities of patients were ascertained utilizing the Charlson comorbidity index, which is computed utilizing the ICD-10 codes. The following information was gathered: length of hospitalization (LOS; days), surgical procedures performed during hospitalization, admission to the intensive care unit (ICU), utilization of mechanical ventilatory support, extracorporeal membrane oxygenation, experience with cardiopulmonary resuscitation, nasal or mask oxygen therapy, and extracorporeal membrane oxygenation. Additionally, survivors were categorized into three distinct categories based on the type of healthcare facility they were admitted to as a result of the COVID-19 pandemic: long-term facility care centers, tertiary general hospitals, and general hospitals. The comprehensive expenses incurred for hospitalization, encompassing self-payment as well, were documented in United States Dollars (USD) using a 1200 Won (Korean currency) to 1 USD conversion ratio.

### 2.6. Statistical Analysis

The clinicopathologic attributes of patients are delineated as numerical values accompanied by percentages for categorical and continuous variables, respectively, and mean values accompanied by the standard deviation (SD). Clinicopathologic characteristics of the QOL worsening group were compared to those of the group that did not experience any QOL worsening using the chi-square test for categorical variables and the *t*-test for continuous variables. An additional approach was to develop a multivariable logistic regression model to analyze the independent associated factors contributing to the decline in quality of life among COVID-19 survivors. Incorporating each covariate into the adjusted model was done. However, in the multivariable model, QOL-related factors such as household income, disability, and employment status at the time of hospital admission due to COVID-19 were excluded. This was because the primary outcome, which was the deterioration of QOL, was assessed solely through direct comparisons of the QOL factors prior to and subsequent to the onset of COVID-19. The findings are displayed in the form of odds ratios (ORs) accompanied by 95% confidence intervals (CIs). To validate the models’ goodness of fit, the Hosmer–Lemeshow test was employed. Multicollinearity did not exist in the multivariable models due to the fact that the intervariable variance inflation factors were all below 2.0. The R programming language (version 4.0.3; R Foundation for Statistical Computing, Vienna, Austria) was utilized to conduct every statistical analysis. The threshold for statistical significance was *p* < 0.05.

## 3. Results

Table 1 shows the clinicopathological characteristics of COVID-19 survivors. A total of 106,091 (32.6%) survivors experienced QOL worsening after COVID-19. Specifically, 21,223 (6.5%) participants experienced job loss, 94,556 (29.0%) had decreased household income, and 559 (0.2%) had newly acquired disabilities.

Table 2 shows the results of the comparison of the clinicopathological characteristics between COVID-19 survivors in the QOL worsening and no QOL worsening groups. The mean age was higher in the QOL worsening group than in the no QOL worsening group (*p* < 0.001), and the proportion of ICU admissions was higher in the QOL worsening group than in the no QOL worsening group (*p* = 0.01)

### QOL Worsening

Table 3 shows the QOL results before and after COVID-19 among survivors. After COVID-19, the proportions of COVID-19 survivors in the unemployment group, medical aid and Q1 (in the lowest) groups of household income, and mild-to-moderate disability group were higher than the proportions recorded in the year of COVID-19 diagnosis.

Table 4 shows the results of the multivariable logistic regression analysis of QOL worsening after COVID-19 among survivors. Living in rural areas (*p* = 0.009), ICU admission (*p* = 0.03), and increase in self-payment by 100 USD (*p* < 0.001) were associated with increased QOL worsening after COVID-19. Old age (*p* < 0.001), first vaccination (*p* < 0.001), and second vaccination (*p* < 0.001) were associated with decreased QOL worsening after COVID-19.

## 4. Discussion

This population-based cohort study showed that approximately one-third of COVID-19 survivors admitted to hospitals or monitoring centers in South Korea experienced QOL worsening by 31 March 2022. Some factors, such as ICU admission, younger age, non-vaccination, increased self-payment for treatment, and cost of hospitalization, were identified to be associated with QOL worsening. As previous studies have focused on QOL worsening among COVID-19 survivors regarding social function, physical role, mental health, pain, activity change, and discomfort [11], our study focused on socioeconomic factors such as unemployment, decreased household income, and newly acquired disability. Moreover, we identified potential risk factors for QOL worsening among survivors, such as living in rural areas, ICU admission, increased self-payment, and non-vaccination. This is the novelty of this study.

Decreased household income levels and unemployment among COVID-19 survivors show that COVID-19 is a critical public health issue in endemic situations. In the United States, an unemployment crisis with massive job losses was reported with the initial wave of COVID-19 occurring in 2020 [15]. Unemployment has been considered a challenging issue in estimating the total lives lost due to COVID-19-related decisions [16]. A recent cohort study also reported that COVID-19 survivors who have lost their jobs have worse mental health status and depression levels [17].

In a national survey conducted in China between 20 March 2020 and 29 April 2020, almost half (47.51%) of the respondents reported partial income loss—that is, a somewhat lower income than their income before the COVID-19 outbreak [18]. Similar results were reported in Malaysia during the COVID-19 pandemic lockdown [19]. Our results are valuable from two perspectives. First, most previous studies have focused on household income loss using surveys [18,19], which may have introduced biases such as non-response, response, selection, and volunteer bias [20]. However, our study used data from the NHIS database, which contains the annual household income of all individuals, to determine insurance premiums in the year as the sole public health insurance in South Korea. Therefore, accurate decreases in household income could be evaluated among COVID-19 survivors in this study. Second, household income in the NHIS database reflects both the income of employees and self-employees, in addition to the properties of the unemployed. Therefore, the economic status of all COVID-19 survivors could be evaluated in detail.

Previous literature has reported that impaired physical function is a critical factor for QOL among COVID-19 survivors [21,22]. In this study, some COVID-19 survivors acquired new disabilities after COVID-19. We examined the proportion of newly acquired disabilities in detail, and the most common newly acquired disability was brain disability, suggesting that many survivors suffered from shock or hypoxic brain damage. Moreover, maintaining daily life is crucial when disability occurs, because disability signifies a severe physical impairment associated with COVID-19.

Another important finding was that both the first and second vaccinations were associated with decreased QOL worsening among COVID-19 survivors. It is well known that patients hospitalized for COVID-19 are less likely to have received vaccination for COVID-19, and they mostly require mechanical ventilation or their condition mostly progress to death [23,24]. Therefore, it is possible that COVID-19 survivors who received vaccination may have experienced a less severe form of COVID-19, resulting in less QOL worsening. Similarly, ICU admission was a potential risk factor for QOL worsening among the COVID-19 survivors in this study. ICU admission suggested that COVID-19 survivors suffered a severe form of COVID-19, which might have caused a sequalae resulting in increased QOL worsening.

An increased self-payment amount was associated with increased QOL worsening among COVID-19 survivors. In South Korea, the government pays for the entire cost of treatment for patients diagnosed with COVID-19, as it is included in the insurance scheme to allow the hospitalization of patients with financial challenges, though some non-insured treatments are excluded. Although the treatment for the majority of hospitalizations associated with COVID-19 are covered by the government, partial self-payment could have been a risk factor for QOL worsening among COVID-19 survivors. Our results showed that a 100 USD increase in self-payment was associated with a 2% increase in QOL worsening, which may have had a small impact on QOL worsening among COVID-19 survivors.

This study has several limitations. First, we did not evaluate long-term QOL worsening among COVID-19 survivors, because the KDCA-NHIS database provided follow-up data until 31 March 2022. There might be bias, because patients with COVID-19 have different recovery times, and their health status may be different. Moreover, QOL worsening among COVID-19 survivors may be ongoing, and more studies are needed to address this issue in the future. Second, we did not consider the types of COVID-19 because of a lack of this information in the database. As various types of COVID-19, such as the omicron, delta, and alpha variants, have different clinical severities and responses to vaccination [25,26], QOL worsening among COVID-19 survivors may be affected. Third, because medical and social infrastructures vary by country, the conclusions of this study may not be generalizable to different countries. Fourth, because the primary outcome (QOL deterioration) was evaluated by direct comparisons of the QOL characteristics of participants before and after COVID-19, we did not incorporate QOL-related covariates (employment status, household income, and disability) at hospital admission in the analysis. This may have introduced bias, as the participants could not be identified as having lost work or having decreased household income following COVID-19; patients who were unemployed or on the medical aid program had a low risk of QOL worsening. As we did not perform Cox regression analysis, we could not establish the timing of exposure (variables) or outcome (QOL worsening) occurrence. Furthermore, we could not use time-to-event analysis to determine the timeframe from hospital admission to QOL worsening, because the primary outcome (QOL deterioration) for all COVID-19 survivors was assessed concurrently on 31 March 2022. In addition, the inability to assess the period between the COVID-19 survivors’ discharge time and QOL deterioration may have introduced bias. Therefore, the findings of this study should be interpreted with caution. Future research should use time-to-event analysis and long-term follow-up to confirm our study findings.

## 5. Conclusions

In conclusion, in South Korea, approximately one-third of COVID-19 survivors admitted to hospitals or monitoring centers experienced QOL worsening. Some potential risk factors for QOL worsening were identified. Our results suggest that hospitalized COVID-19 survivors need social policy support that considers unemployment, lost earnings, and newly acquired disability after discharge from the hospital. Moreover, it is important for hospitalized COVID-19 survivors to be supported to improve their QOL after hospital discharge, as well as return to their daily life routine before their COVID-19 diagnosis.

## Figures and Tables

**Table 1:** Clinicopathological characteristics of adult COVID-19 survivors admitted to hospitals or monitoring centers in South Korea from 8 October 2020 to 31 December 2021.

Variable		Mean (SD)	*N* (%)
Age		49.1 (17.9)	
Male sex			166,073 (51.0)
Residence			
	Urban area		161,849 (49.7)
	Rural area		163,817 (50.3)
Infection route			
	Domestic		320,386 (98.4)
	Foreign		5280 (1.6)
Cause type			
	Inflow from foreign countries		5280 (1.6)
	Contact with person-related inflow from foreign countries		143 (0.0)
	Outbreak in hospitals or nursing care centers		16,377 (5.0)
	Outbreak in local communities		69,442 (21.3)
	Contact with a COVID-19 confirmed patient		134,191 (41.2)
	Unknown		100,233 (30.8)
CCI, point		2.6 (2.6)	
LOS, day		10.0 (4.1)	
Receipt of surgery during hospitalization			1295 (0.4)
Type of hospital			
	Tertiary general hospital		33,074 (10.2)
	General hospital		283,556 (87.1)
	Long-term facility care center		9036 (2.8)
1st vaccination			287,804 (88.4)
2nd vaccination			271,179 (83.3)
ICU admission			4930 (1.5)
Nasal or mask oxygen therapy			28,584 (8.8)
Mechanical ventilator support			528 (0.2)
ECMO support			50 (0.0)
Experience of CPR			
CRRT use			6 (0.0)
Total cost for hospitalization in USD		1483.2 (2435.8)	
	Self-payment in USD	222.3 (383.6)	
Total QOL worsening group			106,091 (32.6)
	Unemployment group		21,223 (6.5)
	Decreased household income group		94,556 (29.0)
	Newly acquired disability group		559 (0.2)

COVID-19, coronavirus disease 2019, SD, standard deviation; CCI, Charlson comorbidity index; LOS, length of hospital stays; ICU, intensive care unit; ECMO, extracorporeal membrane oxygenation; CPR, cardiopulmonary resuscitation; CRRT, continuous renal replacement therapy; USD, United States Dollars; QOL, quality of life.

**Table 2:** Comparison of the clinicopathological characteristics between COVID-19 survivors in the QOL worsening and no QOL worsening groups admitted to hospitals or monitoring centers in South Korea from 8 October 2020 to 31 December 2021.

Variable		QOL Worsening Group *n* = 106,091	No QOL Worsening Group *n* = 219,575	*p*-Value
Age, year		48.7 (17.8)	49.2 (17.9)	<0.001
Male sex		54,335 (51.2)	111,738 (50.9)	0.08
Residence				0.009
	Urban area	52,374 (49.4)	109,475 (49.9)	
	Rural area	53,717 (50.6)	110,100 (50.1)	
Infection route				
	Domestic	104,384 (98.4)	216,002 (98.4)	
	Foreign	1707 (1.6)	3573 (1.6)	0.70
Cause type				<0.001
	Inflow from foreign countries	1707 (1.6)	3573 (1.6)	
	Contact with person-related inflow from foreign countries	45 (0.0)	98 (0.0)	
	Outbreak in hospitals or nursing care centers	4609 (4.3)	11,768 (5.4)	
	Outbreak in local communities	22,473 (21.2)	46,969 (21.4)	
	Contact with a COVID-19 confirmed patient	44,061 (41.5)	90,130 (41.0)	
	Unknown	33,196 (31.3)	67,037 (30.5)	
CCI, point		2.5 (2.6)	2.6 (2.6)	<0.001
LOS, day		9.9 (4.1)	10.0 (4.2)	0.002
Receipt of surgery during hospitalization		448 (0.4)	847 (0.4)	0.12
Type of hospital				<0.001
	Tertiary general hospital	10,867 (10.2)	22,207 (10.1)	
	General hospital	92,504 (87.2)	191,052 (87.0)	
	Long-term facility care center	2720 (2.6)	6316 (2.9)	
1st vaccination		93,213 (87.9)	194,591 (88.6)	<0.001
2nd vaccination		88,068 (83.0)	183,111 (83.4)	0.006
ICU admission		1690 (1.6)	3240 (1.5)	0.01
Nasal or mask oxygen therapy		9353 (8.8)	19,231 (8.8)	0.21
Mechanical ventilator support		192 (0.2)	336 (0.2)	0.06
ECMO support		20 (0.0)	30 (0.0)	0.26
Experience of CPR		3 (0.0)	9 (0.0)	0.12
CRRT use		3 (0.0)	3 (0.0)	0.75
Self-payment, 100 USD		230.2 (413.5)	218.5 (368.2)	<0.001

COVID-19, coronavirus disease 2019; QOL, quality of life; CCI, Charlson comorbidity index; LOS, length of hospital stays; ICU, intensive care unit; ECMO, extracorporeal membrane oxygenation; CPR, cardiopulmonary resuscitation; CRRT, continuous renal replacement therapy; USD, United States Dollars.

**Table 3:** QOL results before and after COVID-19 among survivors admitted to hospitals or monitoring centers in South Korea from 8 October 2020 to 31 December 2021.

Variable		In the Year When They Were Diagnosed with COVID-19	31 March 2022 (Last Follow-Up Date)
Having a job *		215,894 (66.3)	212,324 (65.2)
Household income level group			
	Medical aid group	11,275 (3.5)	11,830 (3.6)
	Q1 (lowest)	63,196 (19.4)	67,256 (20.7)
	Q2	72,596 (22.3)	66,558 (20.4)
	Q3	78,106 (24.0)	78,514 (24.1)
	Q4 (highest)	95,203 (29.2)	95,169 (29.2)
	Unknown	5290 (1.6)	6339 (1.9)
Disability **			
	Mild to moderate	12,117 (3.7)	12,358 (3.8)
	Severe	7355 (2.3)	7624 (2.3)

* Those who were self-employed numbered 82,754 in the year of COVID-19 diagnosis and 81,652 on 31 March 2022. ** The number of people in the disability group was 19,472 in the year of COVID-19 diagnosis and 19,982 on 31 March 2022. QOL, quality of life; COVID-19, coronavirus disease 2019.

**Table 4:** Multivariable logistic regression analysis of QOL worsening after COVID-19 among survivors admitted to hospitals or monitoring centers in South Korea from 8 October 2020 to 31 December 2021.

Variable		OR (95% CI)	*p*-Value
Age, 10 years		0.99 (0.98, 0.99)	<0.001
Male sex		1.00 (0.99, 1.02)	0.68
Residence			
	Urban area	1	
	Rural area	1.02 (1.01, 1.04)	0.009
Infection route			
	Domestic	1	
	Foreign	0.96 (0.90, 1.02)	0.17
Cause type			
	Inflow from foreign countries	1	
	Contact with person-related inflow from foreign countries	0.00 (0.00-)	
	Outbreak in hospitals or nursing care centers	0.93 (0.65, 1.32)	0.68
	Outbreak in local communities	0.82 (0.79, 0.85)	<0.001
	Contact with a COVID-19 confirmed patient	0.97 (0.95, 0.99)	0.002
	Unknown	0.99 (0.97, 1.01)	0.31
CCI, point		1.00 (1.00, 1.00)	0.52
LOS, day		0.99 (0.99, 1.00)	0.11
Receipt of surgery during hospitalization		1.01 (0.99, 1.02)	0.37
Type of hospital			
	Tertiary general hospital	1	
	General hospital	1.00 (0.98, 1.03)	0.83
	Long-term facility care center	0.95 (0.90, 1.00)	0.04
1st vaccination		0.89 (0.86, 0.93)	<0.001
2nd vaccination		0.95 (0.93, 0.96)	<0.001
ICU admission		1.08 (1.02, 1.15)	0.03
Nasal or mask oxygen therapy		1.02 (0.99, 1.05)	0.30
Mechanical ventilator support		1.06 (0.86, 1.31)	0.58
ECMO support		1.11 (0.61, 2.03)	0.74
Experience of CPR		0.60 (0.16, 2.25)	0.45
CRRT use		1.65 (0.32, 8.41)	0.55
Self-payment, 100 USD		1.02 (1.02, 1.02)	<0.001

COVID-19, coronavirus disease 2019; QOL, quality of life; OR, odds ratio; CI, confidence interval; CCI, Charlson comorbidity index; LOS, length of hospital stays; ICU, intensive care unit; ECMO, extracorporeal membrane oxygenation; CPR, cardiopulmonary resuscitation; CRRT, continuous renal replacement therapy; USD, United States Dollars.

## Data Availability

The datasets generated and analyzed during the current study are available after approval of the South Korean National Health Insurance Service. If someone wants to request the data/or have queries from this study, contact the corresponding author (In-Ae Song, e-mail: songficu@outlook.kr).

## Supplementary Materials

The following supporting information can be downloaded at https://www.mdpi.com/article/10.3390/jpm14060569/s1: Table S1. The ICD-10 codes used by comorbidity to compute the Charlson comorbidity index.
